# Supplementary figures and images for: Death receptor 6 does not regulate axon degeneration and Schwann cell injury responses during Wallerian degeneration
Source: eLife. 2026 Mar 27;14:RP108389. doi: 10.7554/eLife.108389 (PMC13030889; doi:10.7554/eLife.108389)

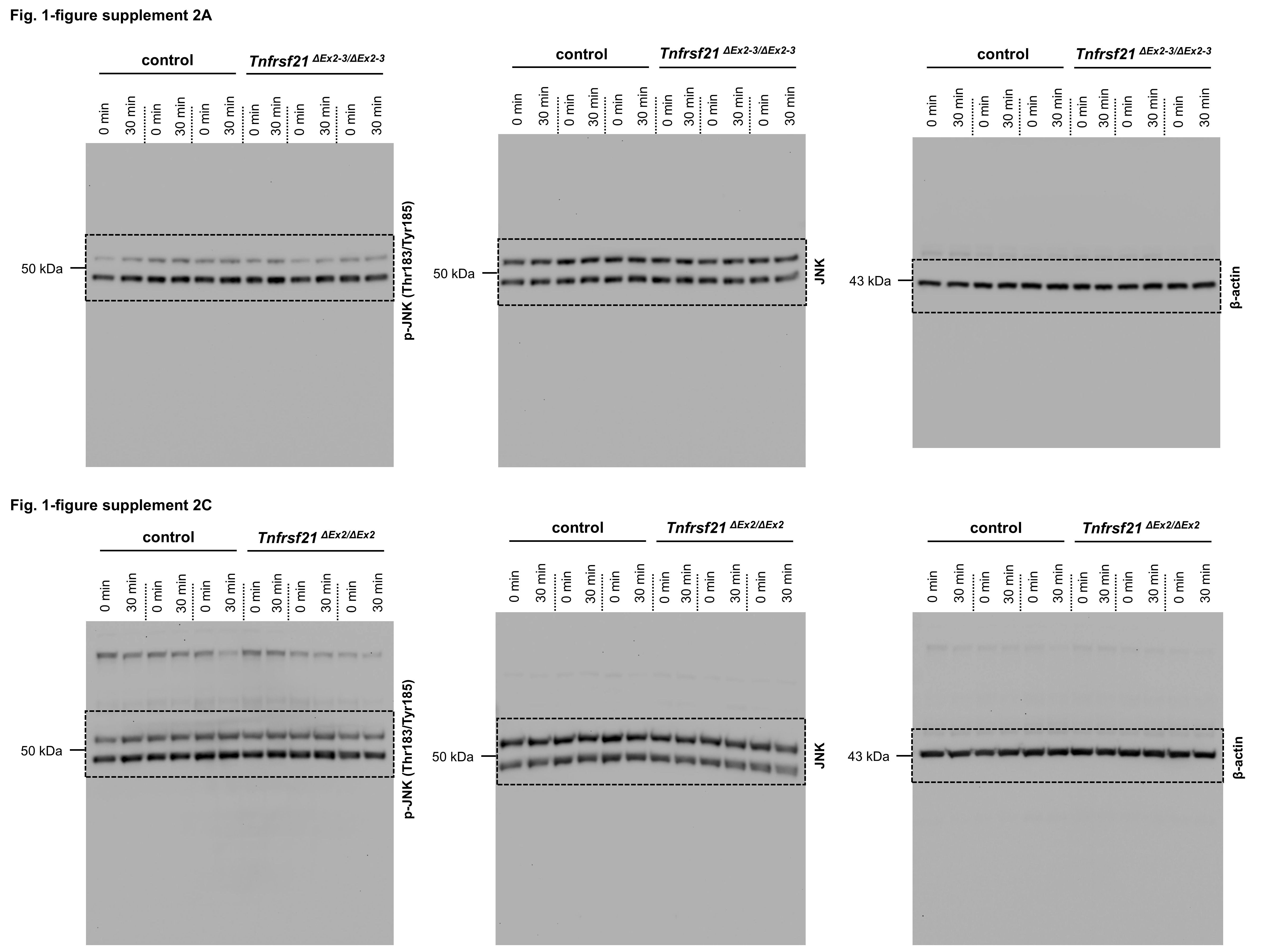

Supplement: Figure 1—figure supplement 2—source data 2. [file elife-108389-fig1-figsupp2-data2.zip › Fig. 1-figure supplement 2-source data 2/Fig. 1-figure suppl 2-source data 2.tif]

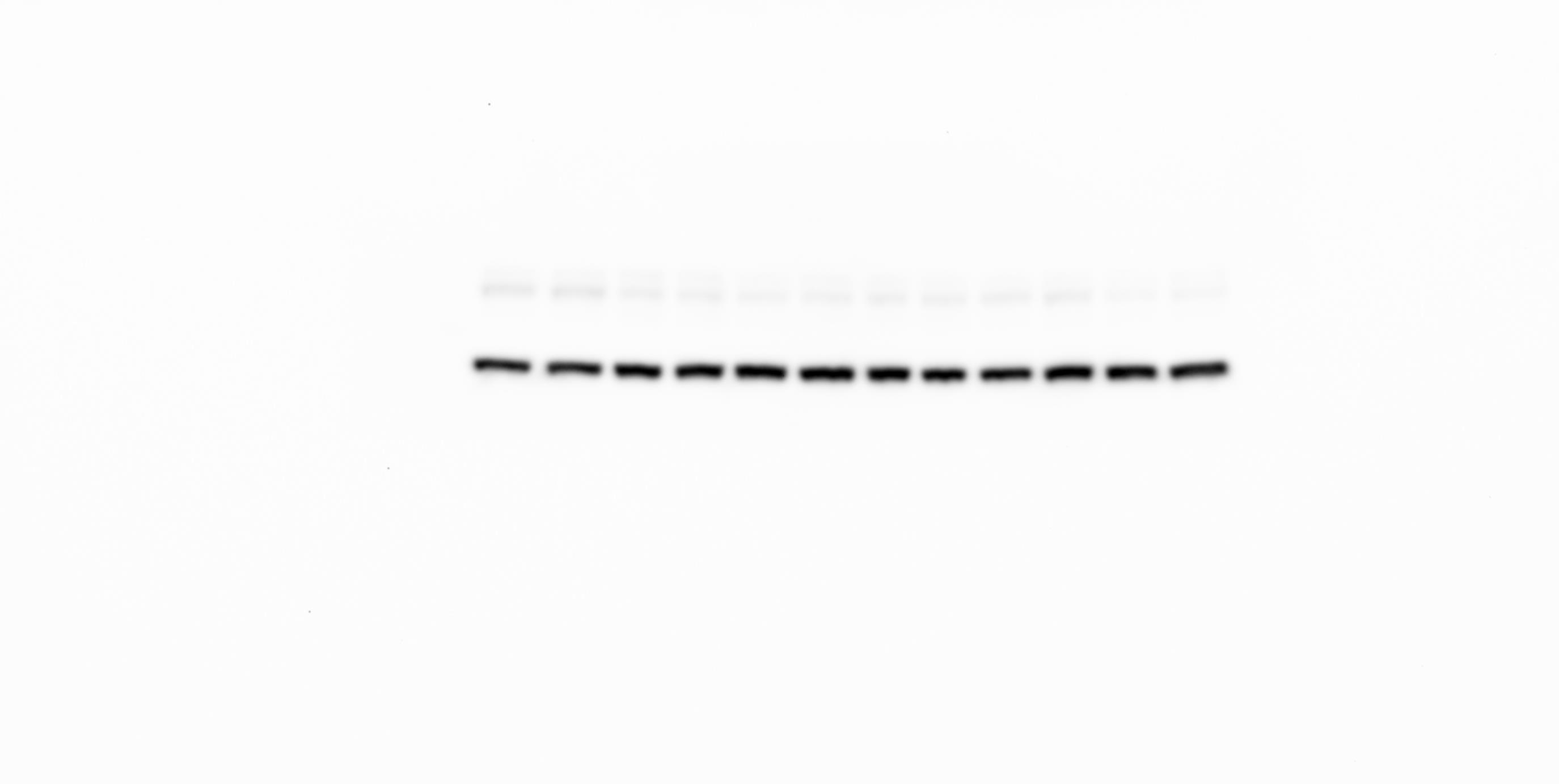

Supplement: Figure 1—figure supplement 2—source data 3. [file elife-108389-fig1-figsupp2-data3.zip › Fig. 1-figure supplement 2-source data 3/Fig. 1-figure supplement 2A beta-actin.tif]

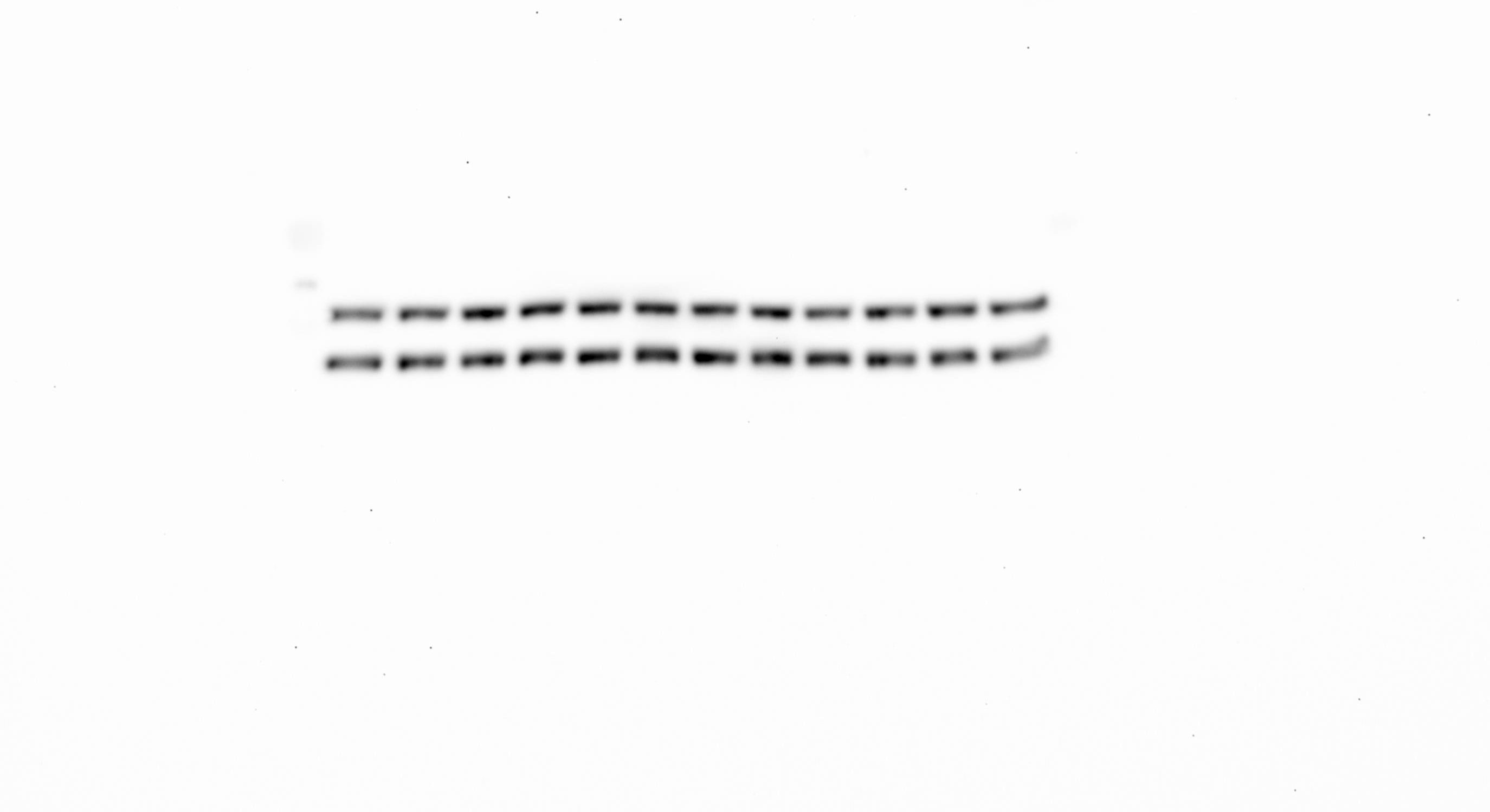

Supplement: Figure 1—figure supplement 2—source data 3. [file elife-108389-fig1-figsupp2-data3.zip › Fig. 1-figure supplement 2-source data 3/Fig. 1-figure supplement 2A JNK.tif]

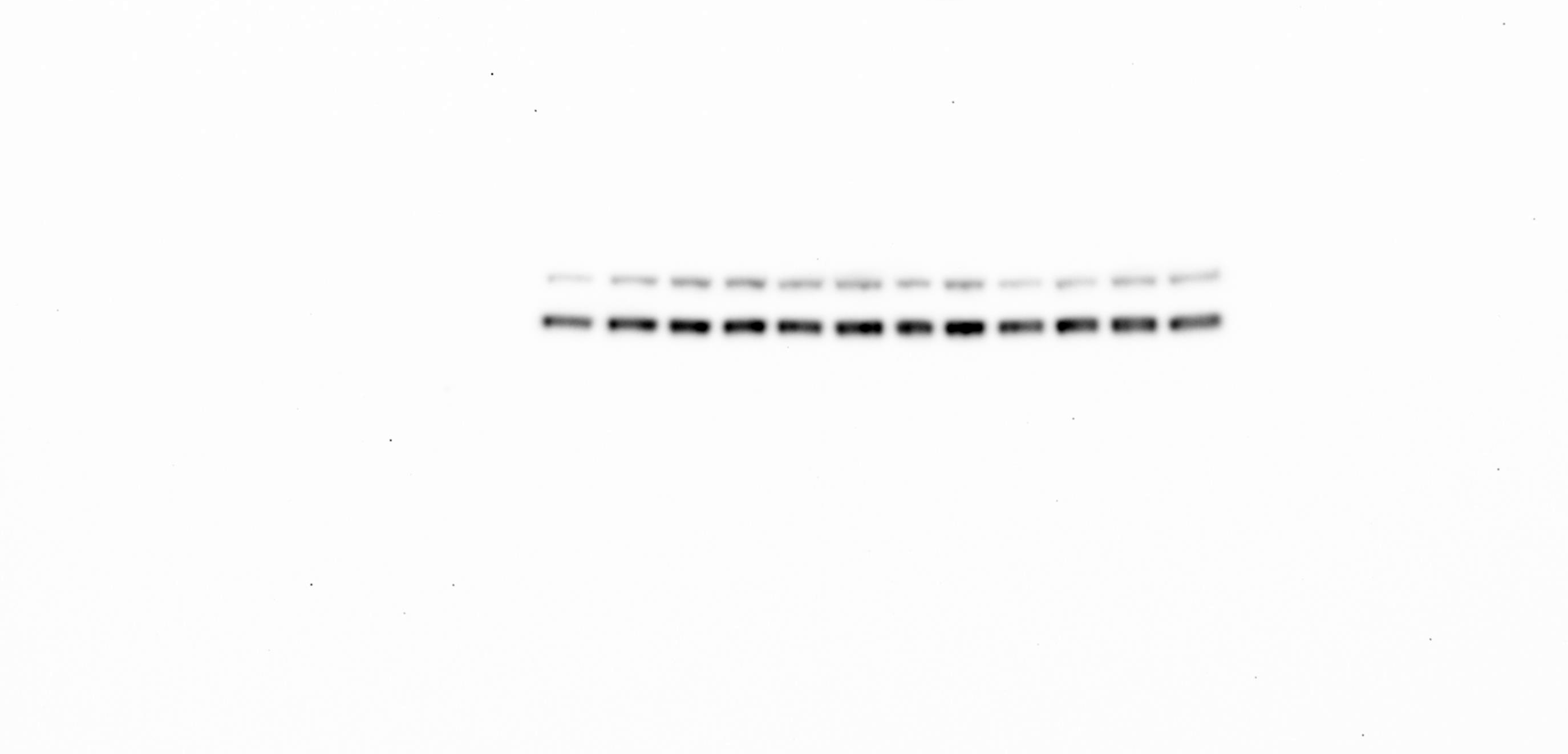

Supplement: Figure 1—figure supplement 2—source data 3. [file elife-108389-fig1-figsupp2-data3.zip › Fig. 1-figure supplement 2-source data 3/Fig. 1-figure supplement 2A p-JNK.tif]

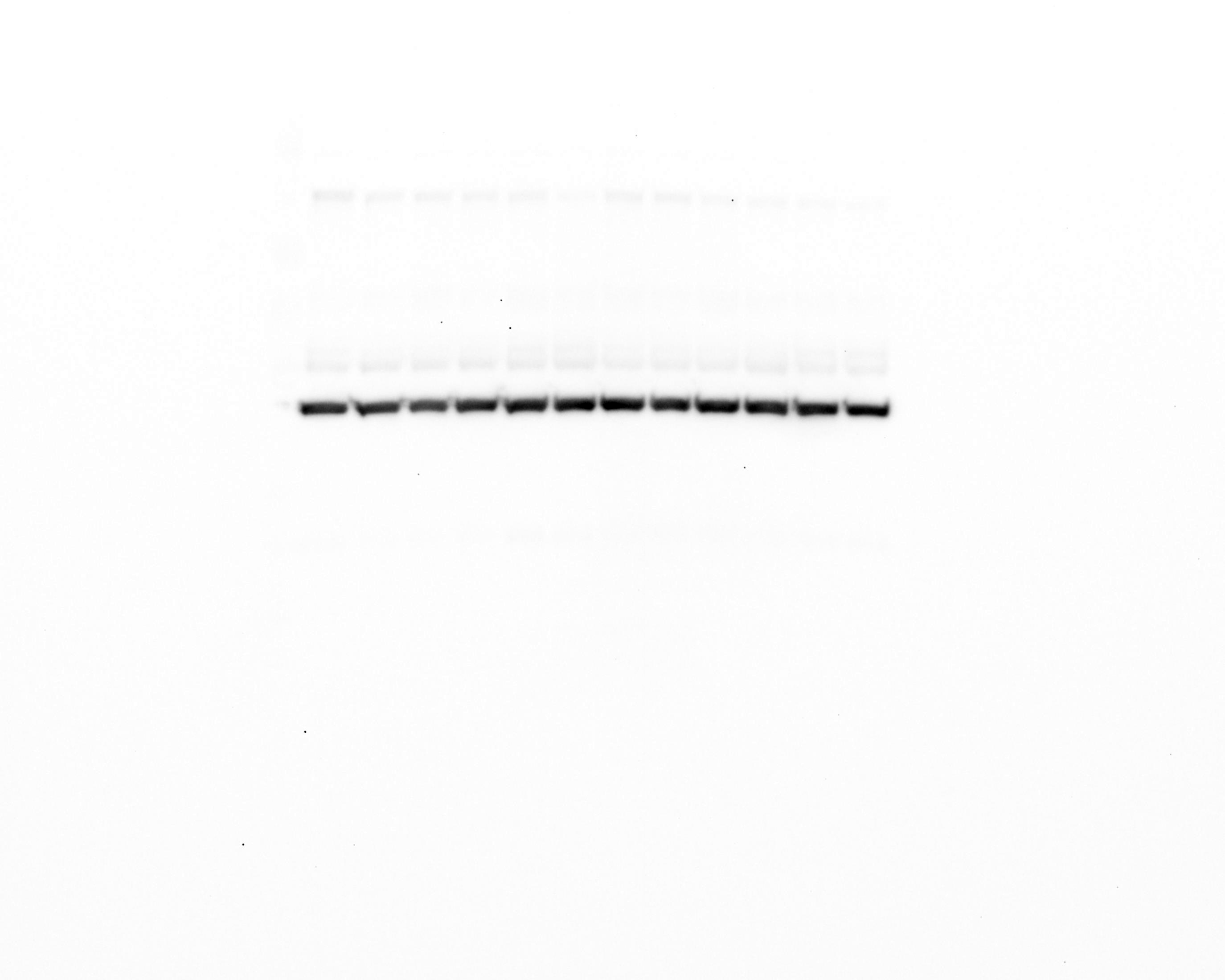

Supplement: Figure 1—figure supplement 2—source data 3. [file elife-108389-fig1-figsupp2-data3.zip › Fig. 1-figure supplement 2-source data 3/Fig. 1-figure supplement 2C beta-actin.tif]

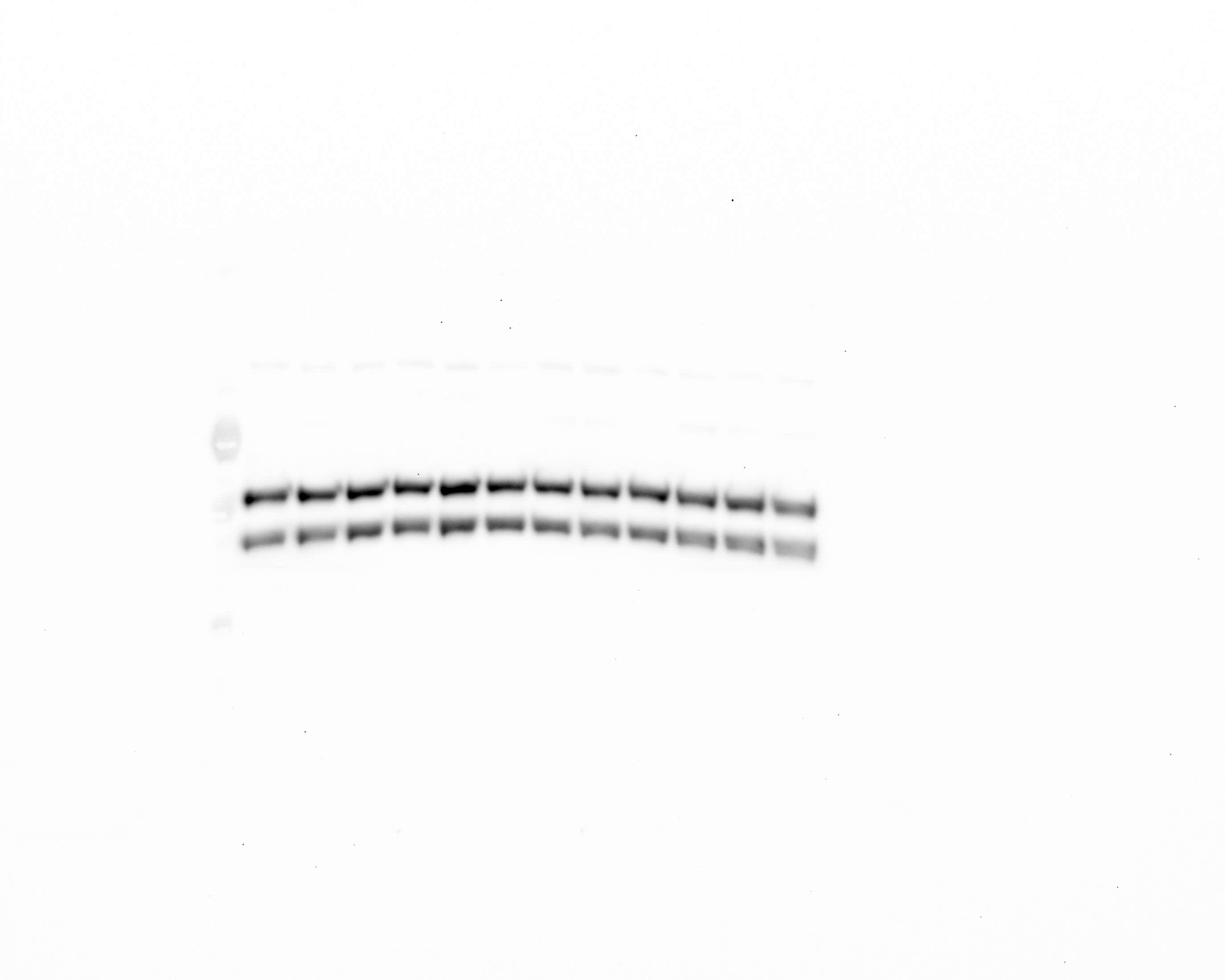

Supplement: Figure 1—figure supplement 2—source data 3. [file elife-108389-fig1-figsupp2-data3.zip › Fig. 1-figure supplement 2-source data 3/Fig. 1-figure supplement 2C JNK.tif]

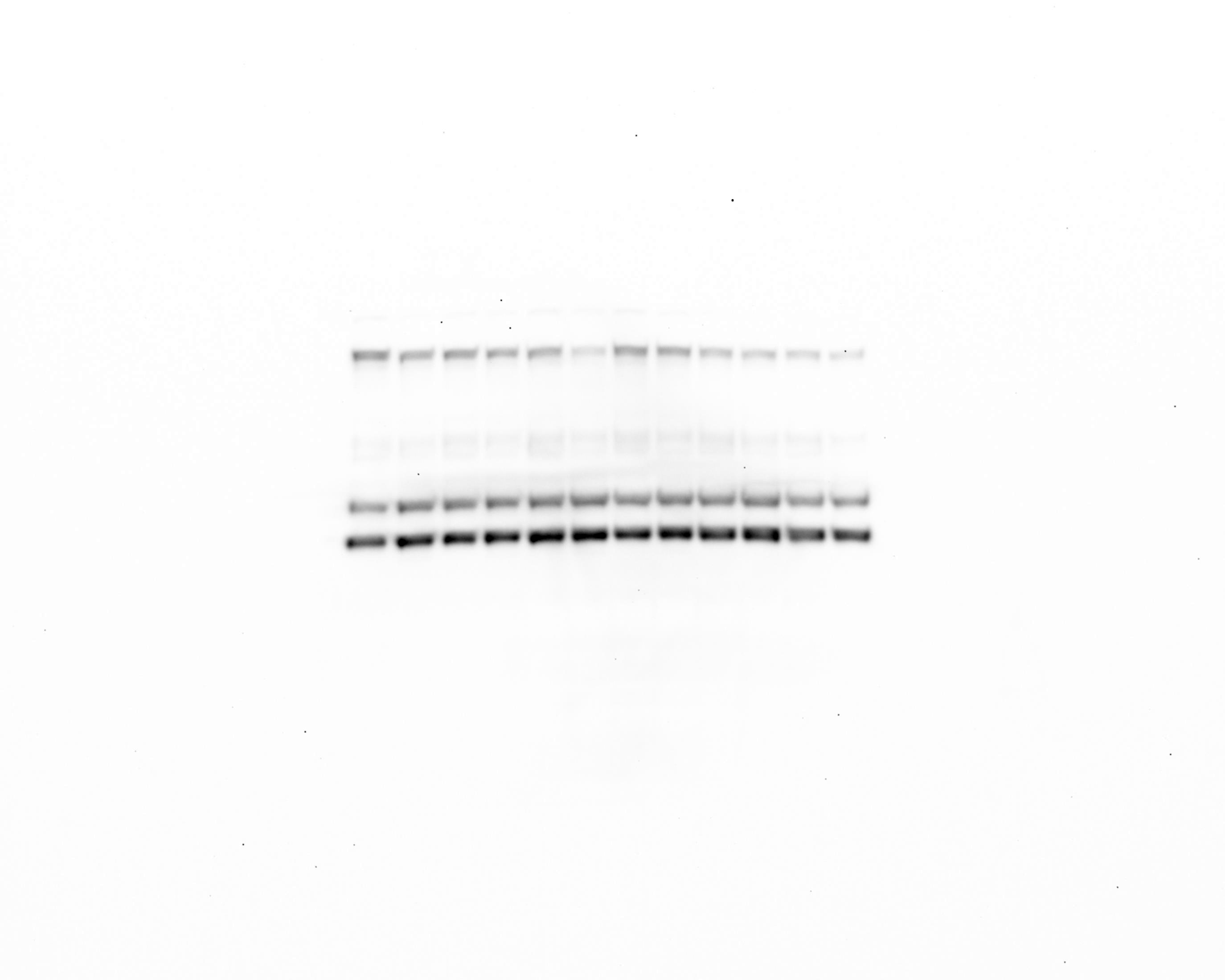

Supplement: Figure 1—figure supplement 2—source data 3. [file elife-108389-fig1-figsupp2-data3.zip › Fig. 1-figure supplement 2-source data 3/Fig. 1-figure supplement 2C p-JNK.tif]

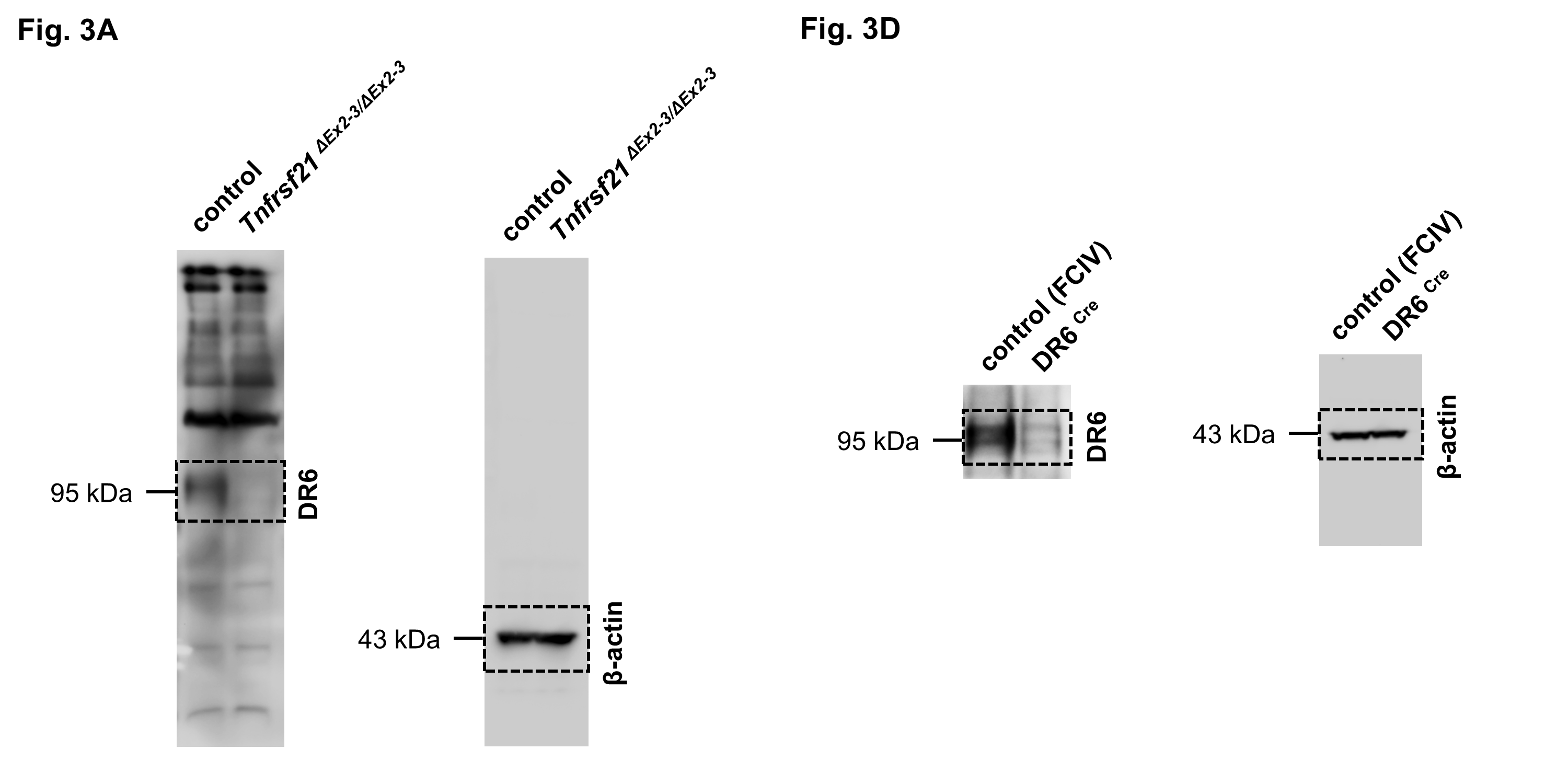

Supplement: Figure 3—source data 2. [file elife-108389-fig3-data2.zip › Fig. 3-source data 2/Fig. 3-source data 2.tif]

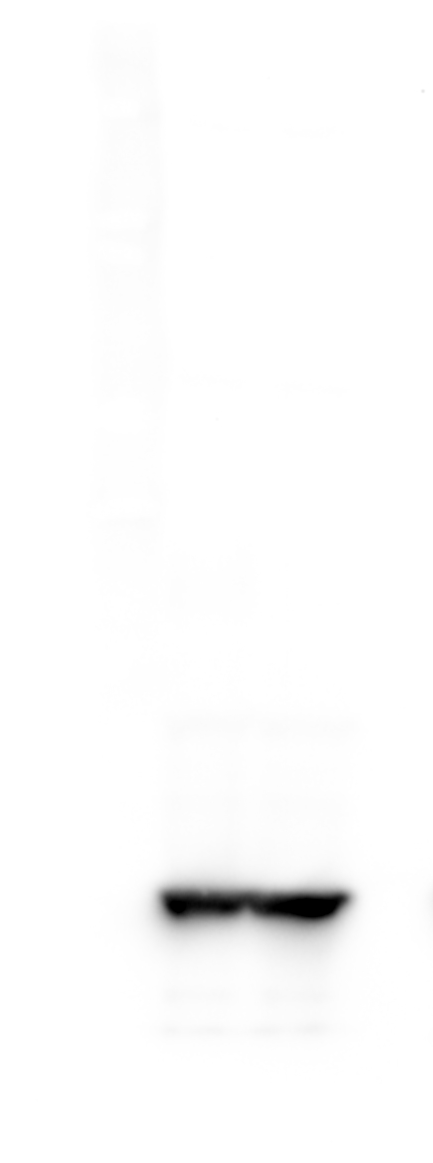

Supplement: Figure 3—source data 3. [file elife-108389-fig3-data3.zip › Fig. 3-source data 3/Fig. 3A beta-actin.tif]

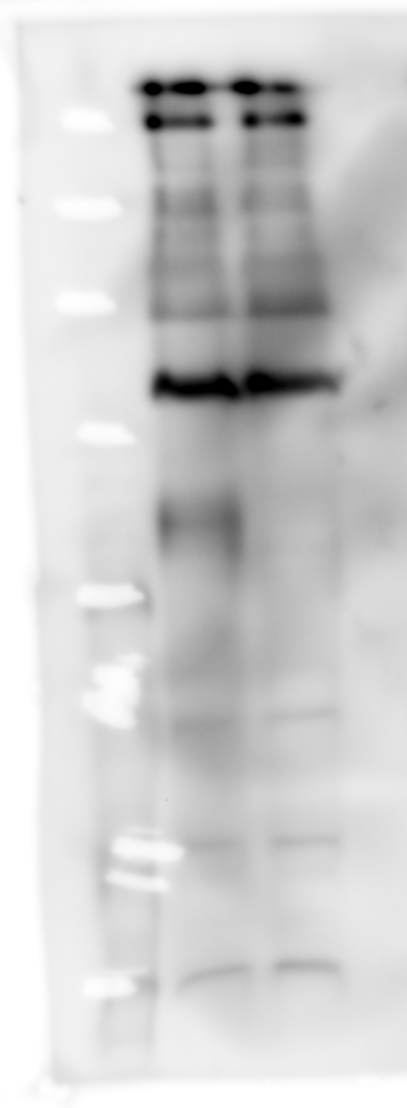

Supplement: Figure 3—source data 3. [file elife-108389-fig3-data3.zip › Fig. 3-source data 3/Fig. 3A DR6.tif]

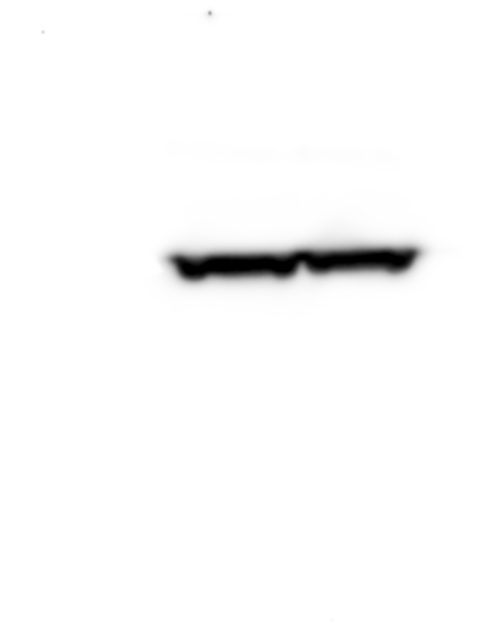

Supplement: Figure 3—source data 3. [file elife-108389-fig3-data3.zip › Fig. 3-source data 3/Fig. 3D beta-actin.tif]

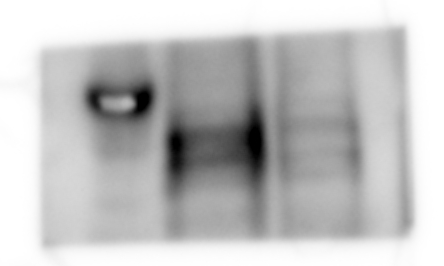

Supplement: Figure 3—source data 3. [file elife-108389-fig3-data3.zip › Fig. 3-source data 3/Fig. 3D DR6.tif]

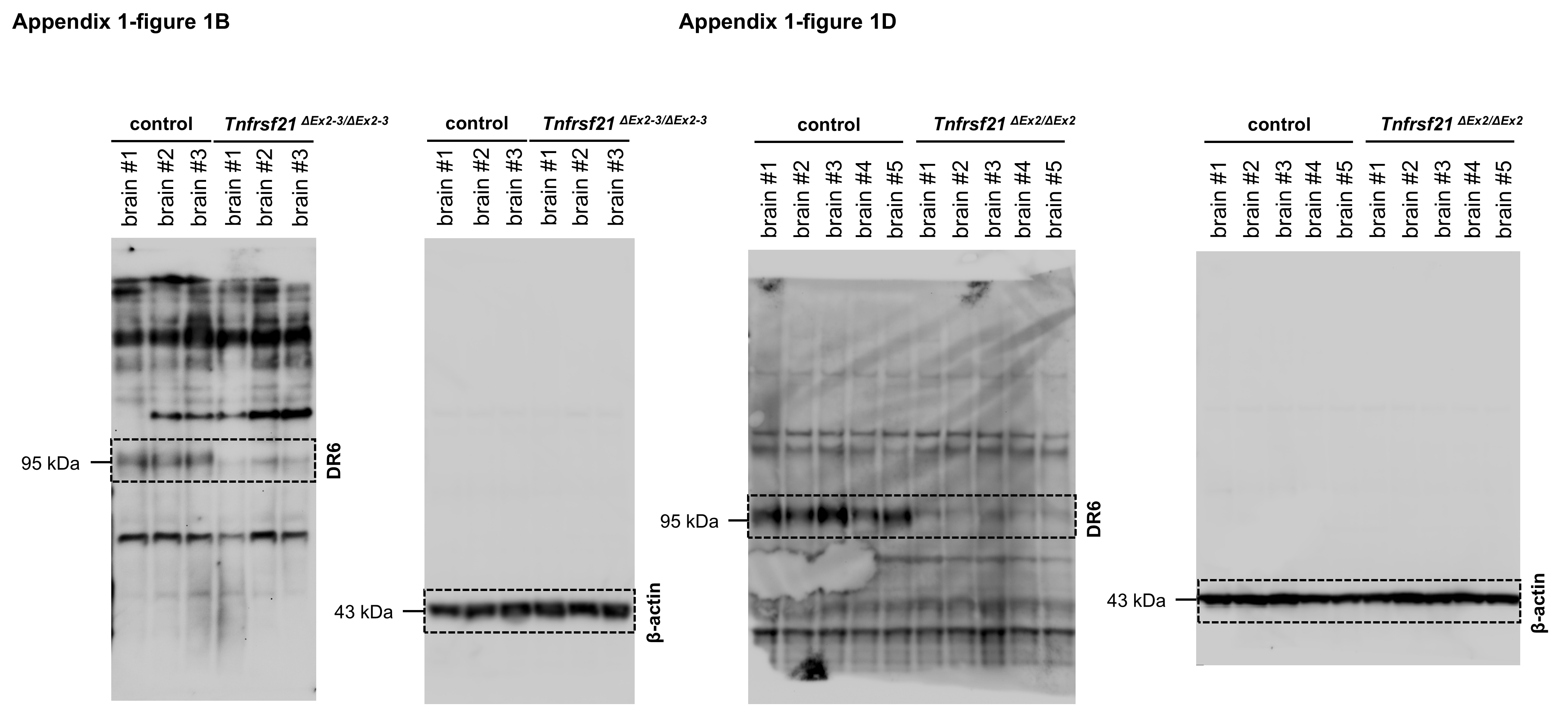

Supplement: Appendix 1—figure 1—source data 2. [file elife-108389-app1-fig1-data2.zip › Appendix 1-figure 1-source data 2/Appendix 1-figure 1-source data 2.tif]

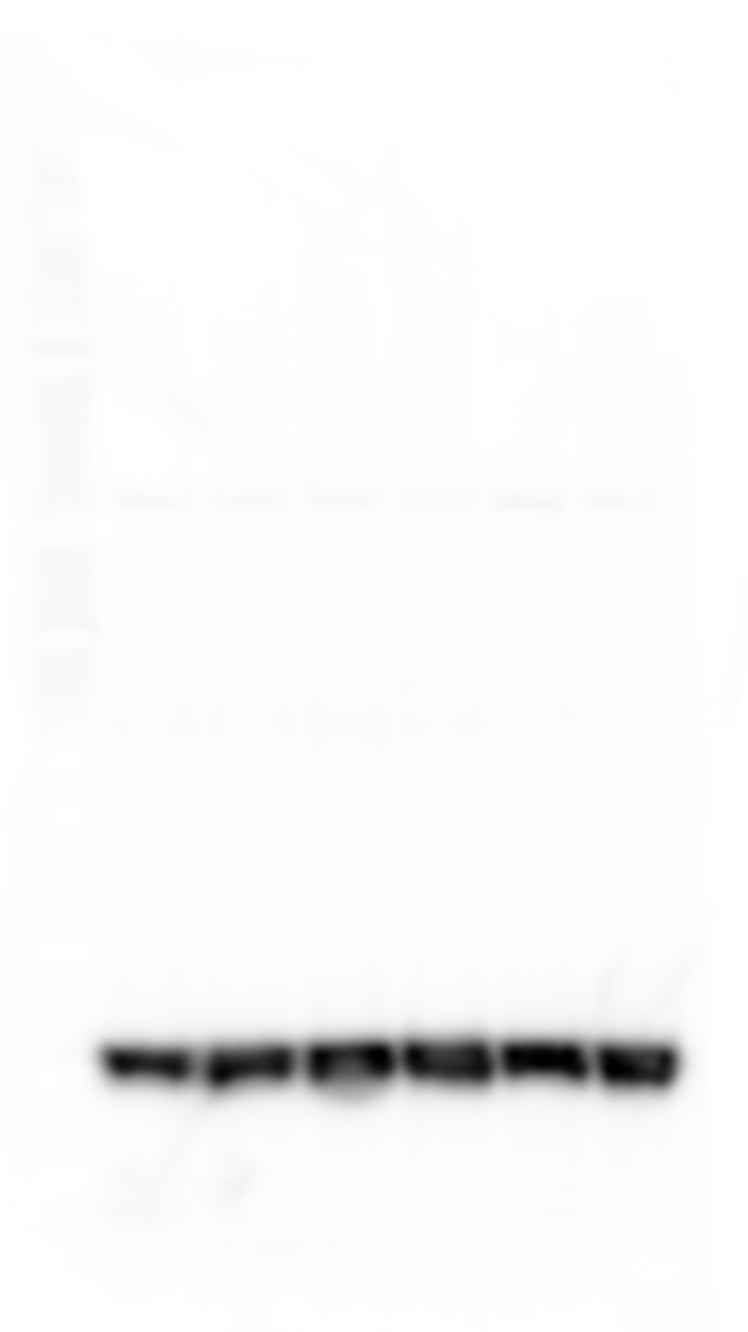

Supplement: Appendix 1—figure 1—source data 3. [file elife-108389-app1-fig1-data3.zip › Appendix 1-figure 1 source data 3/Appendix 1-figure 1B beta-actin.tif]

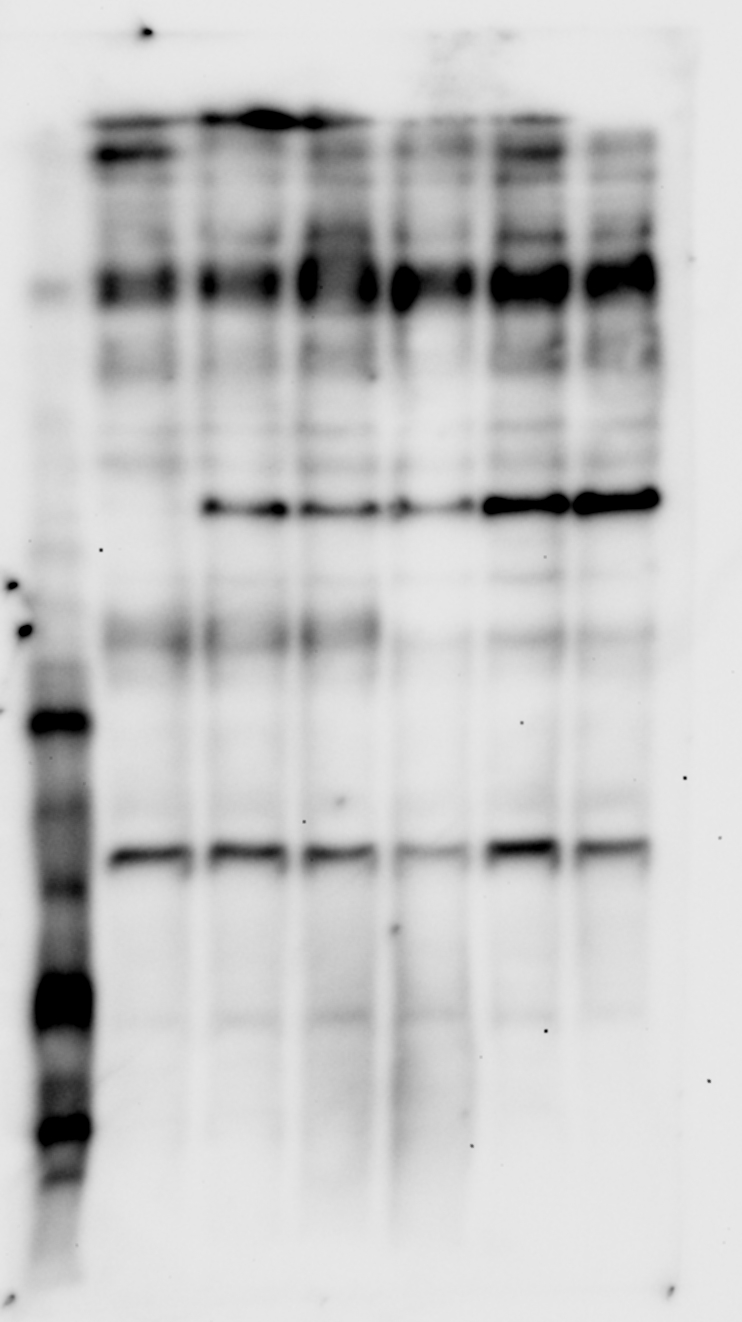

Supplement: Appendix 1—figure 1—source data 3. [file elife-108389-app1-fig1-data3.zip › Appendix 1-figure 1 source data 3/Appendix 1-figure 1B DR6.tif]

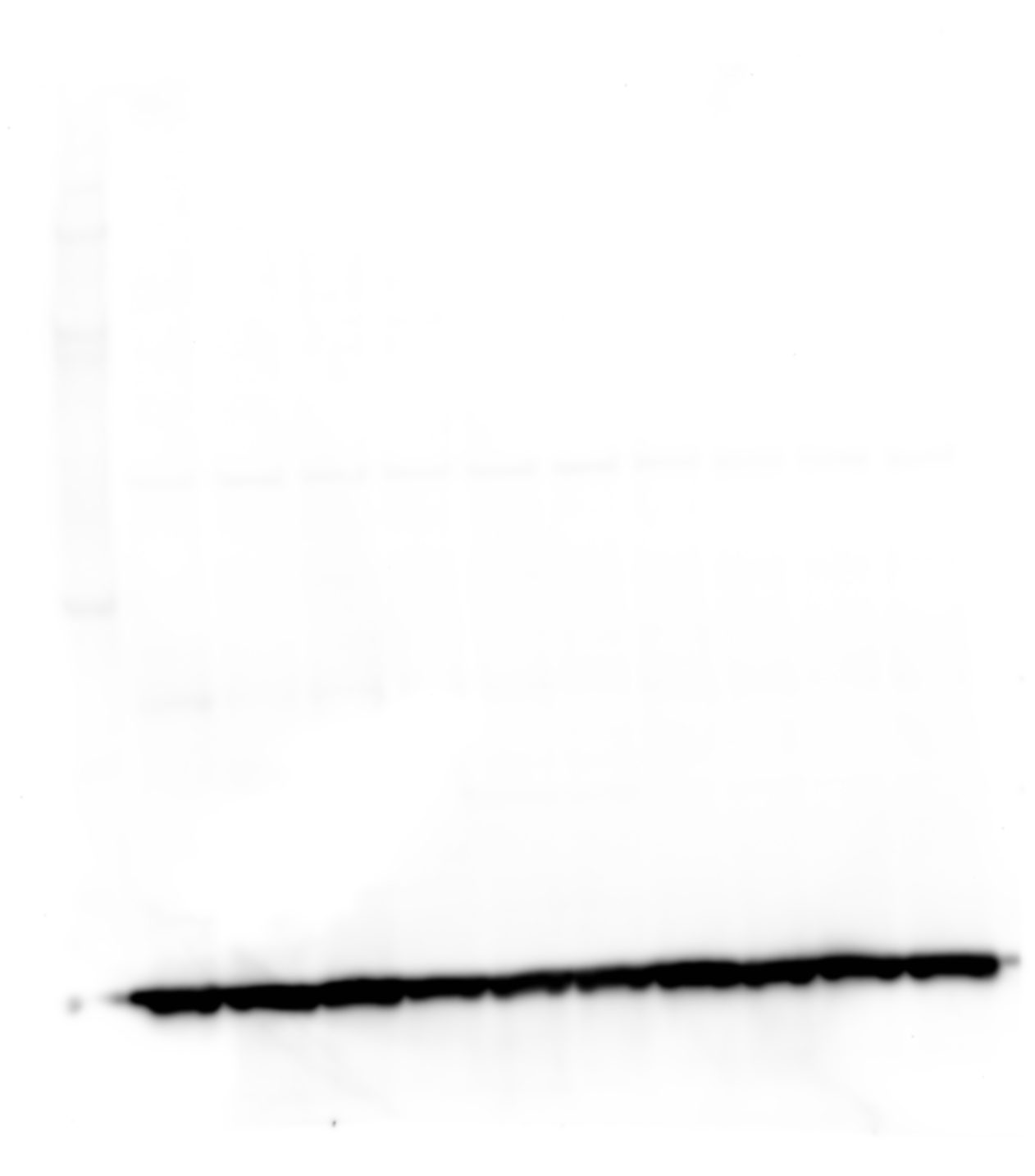

Supplement: Appendix 1—figure 1—source data 3. [file elife-108389-app1-fig1-data3.zip › Appendix 1-figure 1 source data 3/Appendix 1-figure 1D beta-actin.tif]

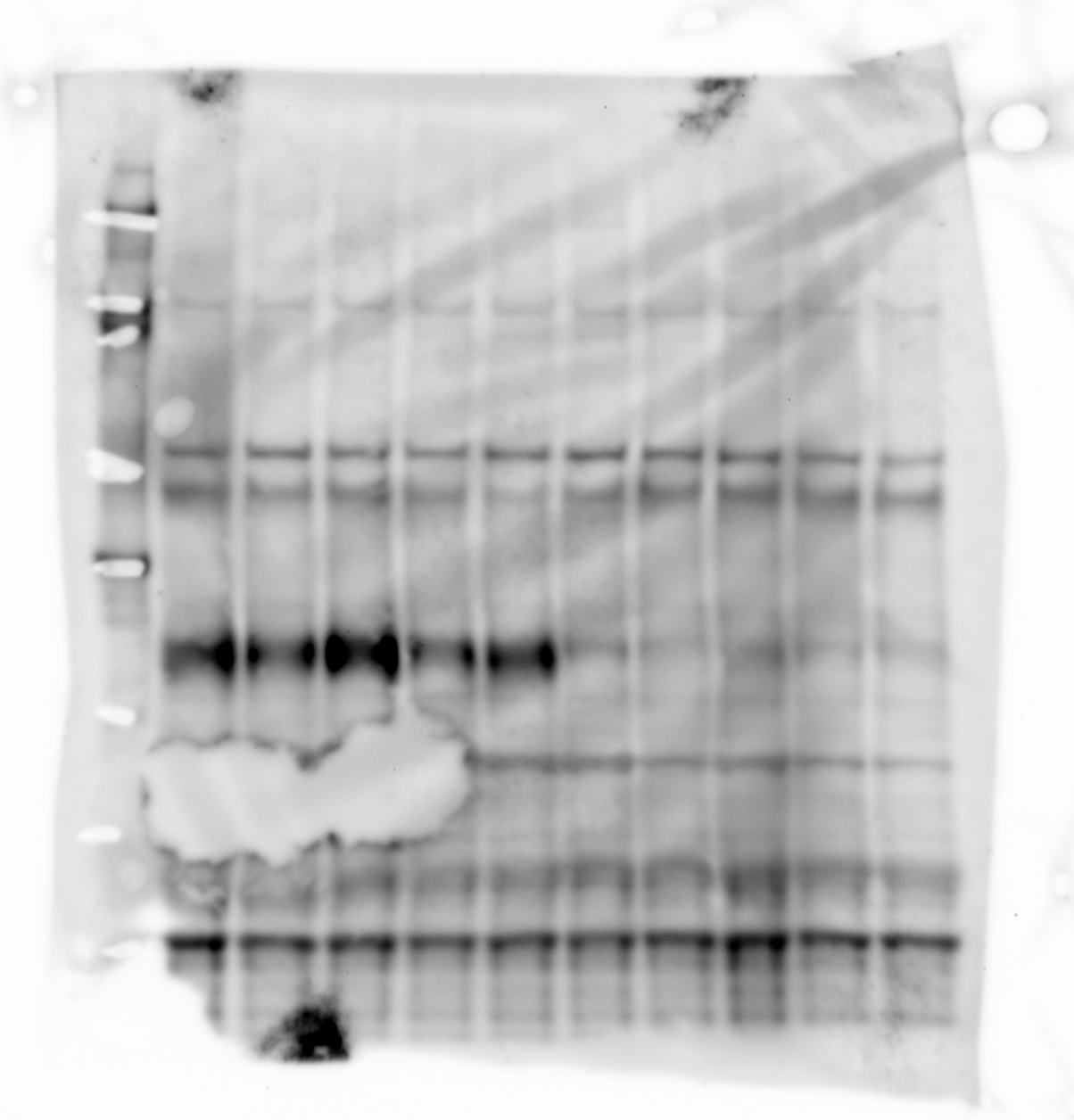

Supplement: Appendix 1—figure 1—source data 3. [file elife-108389-app1-fig1-data3.zip › Appendix 1-figure 1 source data 3/Appendix 1-figure 1D DR6.tif]
